# A Pattern Reconfigurable Quasi-Yagi Antenna Array for 3-D Full-Space Coverage

**DOI:** 10.3390/s25237246

**Published:** 2025-11-27

**Authors:** Ziming Wei, Yihao Xu, Hao Wang, Daolin Fu, Wei Xiong, Yongjin Zhou

**Affiliations:** 1 Shanghai Collaborative Innovation Center of Intelligent Sensing Chip Technology, School of Communication and Information Engineering, Shanghai University, Shanghai 200444, China; zimingwei@shu.edu.cn (Z.W.); xuyihao@shu.edu.cn (Y.X.); 15279101016@163.com (H.W.); 2 Jiangsu Cyberspace Science and Technology Co., Ltd., Nanjing 211111, China; dlfu@fullways.com (D.F.); xiongwei@fullways.com (W.X.)

**Keywords:** pattern reconfigurable antenna, 3-D full-space coverage, unmanned aerial vehicle (UAV)

## Abstract

In this paper, a pattern reconfigurable quasi-Yagi antenna and array for 3-D full-space coverage is proposed. The antenna element consists of hook-shaped radiating patches, parallel-coupled branches, a circular metal patch, and a windmill-shaped metal ground, where four diodes are integrated with the feed lines. By switching the diodes’ operation states, the antenna element can operate in both omnidirectional and directional modes. In the omnidirectional mode, the antenna exhibits a bandwidth of 5.02–5.89 GHz, with a maximum gain of approximately 0.8 dBi. While in the directional mode, the antenna provides a bandwidth of 4.93–5.81 GHz and a maximum gain of 2.4 dBi. In array configuration, directional pattern reconfiguration for azimuth and elevation planes for 3-D full-space coverage can be achieved through excited elements selection and diode states switching, with a peak gain of 4.9 dBi in the directional mode and a pattern non-circularity of 3.1 dB in the omnidirectional mode. The proposed antenna offers advantages such as compact size, light weight, and excellent omnidirectionality, which has great potential in the application of unmanned aerial vehicles (UAVs).

## 1. Introduction

In the context of technological advancements and the progression of modern warfare, unmanned aerial vehicles (UAVs) with low cost, high adaptability, and strong task execution capabilities have been widely deployed in military domains such as reconnaissance, surveillance, and communication [1,2]. As the counter-drone technologies continue to mature, the risks of interference and capture faced by UAVs have significantly increased. At present, UAVs data links mainly employ low-profile omni-directional antennas [3,4,5,6], which offer advantages such as lightweight construction, compact size, aerodynamic shape, and omni-directional radiation characteristics, lacking the anti-interference capabilities. Directional antennas concentrate energy towards the desired target direction, having the potential to improve UAV anti-interference and anti-capture capabilities.

To satisfy diverse communication environments and enhance the survivability of UAVs, it is crucial to design a pattern reconfigurable antenna. Pattern reconfigurable antennas not only retain the advantages of omni-directional communication but also enable seamless switching between omnidirectional and directional modes. Consequently, this technology has attracted considerable research interest due to its ability to enhance UAVs’ resistance against interference and capture. Nowadays, numerous pattern reconfigurable antennas have been proposed [7,8,9,10,11,12,13,14,15]. In Ref. [7], a novel design that incorporates a unique finite periodic structure was proposed. The reconfigurable antenna was capable of steering radiation in four directions without an antenna servo system [8]. To cater to UAVs’ communications, a wideband printed half-bowtie antenna array based on a quad-mode reconfigurable feeding network was presented [9]. However, these solutions generally suffer from large size and high profile. In Ref. [10], a wearable antenna integrated into a military beret for indoor/outdoor positioning was described. The antenna comprises a truncated patch and a circular ring patch, each incorporating four conductive threads, but is constrained by narrow bandwidth. On the other hand, Ref. [11] introduced a compact pattern reconfigurable filtering microstrip antenna with broad bandwidth, though its omnidirectional performance remains limited. From the array synthesis viewpoint, pattern reconfigurable antennas are closely related to classical beamforming and shaped-beam design. In [16], closed-form amplitude distributions for line-source antennas were derived to obtain a narrow main beam with controlled sidelobes. In [17], a deterministic formulation of shaped-beam pattern synthesis was presented for shaped patterns. In [18], robust beam pattern synthesis for antenna arrays with mutual coupling was investigated, showing that optimization techniques can maintain the desired pattern under coupling perturbations. In [19], a general approach to the synthesis of shaped beams for arbitrary fixed-geometry arrays was proposed. These studies provide a theoretical background for understanding and controlling the radiation pattern of antenna arrays beyond simple steering in one plane.

As the application scenarios of communication systems expand from the ground to space, ocean, and multi-node networks, the directional pattern control in a single azimuth plane has become insufficient to meet the demands in complex environments. To enhance the radiation performance and improve the coverage, pattern reconfigurable antennas with 2-D (azimuth and elevation) beam steering capabilities are proposed in the literature [20,21,22,23,24,25,26,27,28,29]. In Ref. [20], a reconfigurable antenna with elevation and azimuth beam switching was presented; the antenna provides beam switching of 65° and 45° in the E-plane and H-plane, respectively. In Ref. [21], a pattern reconfigurable patch antenna with both E-plane and H-plane beam switching based on CSRR-loaded ground was presented; nine different main radiation directions are realized by changing the states of the p-i-n diodes installed on the antenna. Although current research on 2-D beam steering has reached a relatively mature stage, it still faces the critical challenge of being unable to cover the radiation ability in 3-D full-space coverage due to limitations in the beam steering angle range. In addition to these line and planar arrays, recent works have highlighted the importance of array geometry and deterministic synthesis for multi-beam operation. In [30], an unconventional array configuration based on ascending clustered rings was introduced to realize pattern synthesis with a compact structure and simple feeding scheme. In [31], four-beams-reconfigurable circular-ring array antennas for monopulse radar were reported, where a circular geometry is combined with deterministic synthesis to generate several fixed beams in azimuth. In [32], near-field pattern synthesis through spectral factorization and warping strategy was studied, and a deterministic, closed-form approach for pattern control was introduced. These works indicate that, when array geometry and excitation control are jointly designed, compact multi-beam structures suitable for radar and wireless communication links can be realized.

To overcome the above limitations, we propose a pattern reconfigurable antenna to meet the demands of wide bandwidth, 3-D full-space coverage, and good omnidirectionality. For a single element, by controlling the diode’s operating states, the operating mode of the element can be switched from omnidirectional to directional. In the omnidirectional mode, the bandwidth is 5.02–5.89 GHz. The maximum gain is 0.5 dBi, and the pattern non-circularity is less than 2 dB. In the directional mode, the bandwidth is 4.93–5.81 GHz and the maximum gain reaches 2.4 dBi. The overall size is 0.547λ × 0.547λ × 0.027λ. For the array configuration, the 3-D full-space coverage radiation patterns can be realized by both selecting different excitation of the feeding ports and controlling the diode states.

## 2. Antenna Configuration and Working Principle

### 2.1. Antenna Configuration

**Figure 1:**
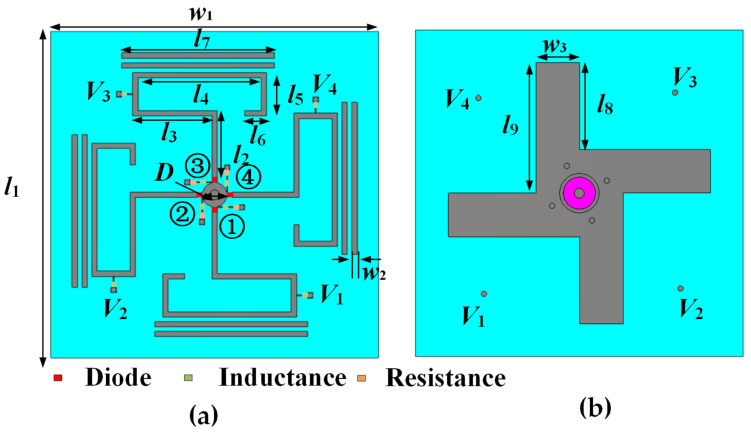
Geometry of the proposed antenna: (**a**) Top view. (**b**) Bottom view.

The configuration of the low-profile pattern reconfigurable antenna is shown in Figure 1. The antenna consists of three layers. The top layer includes a hook-shaped radiating patch, two parallel-coupled branches, a circular metal patch, and the bias network. The hook-shaped metal patch serves as the main radiating element and is fed by a microstrip line. This design reduces the size of the antenna and improves its frequency response and miniaturization level. Two parallel-coupled branches act as directors to guide electromagnetic radiation, while the circular patch enhances the impedance bandwidth. The middle layer is a dielectric substrate made of FR4 with a relative permittivity of 4.4, a loss tangent of 0.02, and a thickness of 1.52 mm. The bottom layer is a windmill-shaped metal ground that functions as the reflector of the Yagi antenna. Diodes are loaded on the feeding line to adjust the operating mode. The dimensions of the antenna are listed in Table 1.

### 2.2. Working Principle

The proposed antenna is a planar printed quasi-Yagi antenna, where the hook-shaped metal patches, coupled branches, and metal ground serve as the active oscillator, director, and reflector, respectively. The planar structure significantly reduces the antenna profile. Compared with traditional Yagi antennas, the design not only has a simple structure but also provides a broad impedance bandwidth. The length of the hook-shaped metal patches is *L* = 0.5*λ_eff_*.

(1) $$L=L_{4}+L_{5}+L_{6}$$

The effective wavelength λ*_eff_* at the resonant frequency is expressed as follows:

(2) $$\lambda_{eff}=\frac{c}{\sqrt{\varepsilon_{eff}}\;f_{r}}$$

where *c* is the speed of light; *f_r_* is the resonant frequency; and *ε_eff_* is the effective dielectric constant.

By controlling the on/off states of the four diodes, the antenna can operate in either omnidirectional or directional modes. The corresponding combinations of the diodes in different states are listed in Table 2. When all diodes are on state, the currents in each element are equal, resulting in omnidirectional radiation. Conversely, when diodes 1 and 2 are on state while diodes 3 and 4 are off state, the current mainly flows through the left and upper elements, producing a directional mode. Utilizing different diode combinations enable flexible beam switching in the azimuth plane.

**Figure 2:**
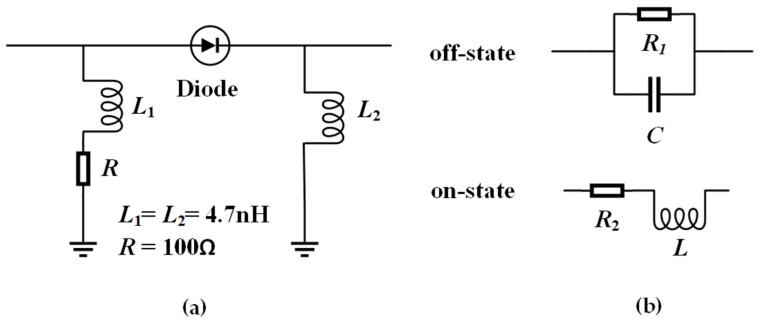
(**a**) Bias circuit. (**b**) Diode equivalent circuit.

The structure of the biasing circuit is illustrated in Figure 2a, which consists of a current-limiting resistor, a diode, and inductors. The model of diode used in this work is MA4AGP907. When the diode is off state, it is modeled as a parallel combination of a resistor *R*_1_ of 300 kΩ and a capacitor *C* of 0.042 pF. On the other hand, when the diode is on state, it is equivalent to a series combination of a resistor *R*_2_ of 4.2 Ω and an inductor *L* of 0.05 nH, as shown in Figure 2b. The equivalent circuit parameters are obtained from the TRL calibration and S-parameter fitting results within the operating frequency band in this work.

## 3. Simulation Results

### 3.1. Single Element

**Figure 3:**
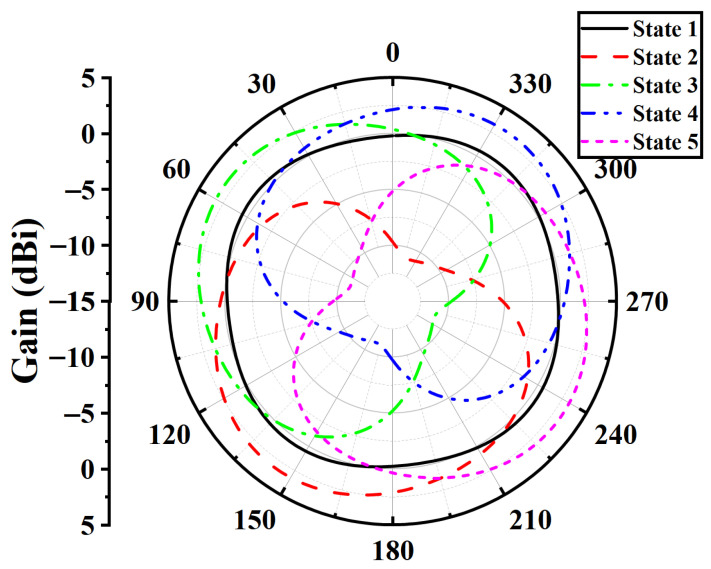
The azimuth radiation patterns of the antenna from State 1 to State 5.

The section presents some simulation results of the single antenna element and analyzes the influence of the coupled branches and the windmill-shaped metal ground on the radiation performance of the antenna. Figure 3 shows the azimuth radiation patterns from State 1 to State 5. In State 1, the antenna operates in omnidirectional mode, and the gain is about 0.8 dBi, whereas in the other states it operates in directional mode with a maximum gain of 3.28 dBi. By selectively biasing the four diodes, the radiation pattern rotates by 90° in each state, resulting in the main lobe of the antenna pointing to 146°, 56°, 326°, and 236°, respectively. The gain obtained when a single diode is switched on is clearly lower than that achieved when two diodes are switched on, and both the radiation efficiency and the directivity are relatively weaker. Therefore, the mode with two diodes switched on simultaneously is adopted as the primary directional radiation mode.

**Figure 4:**
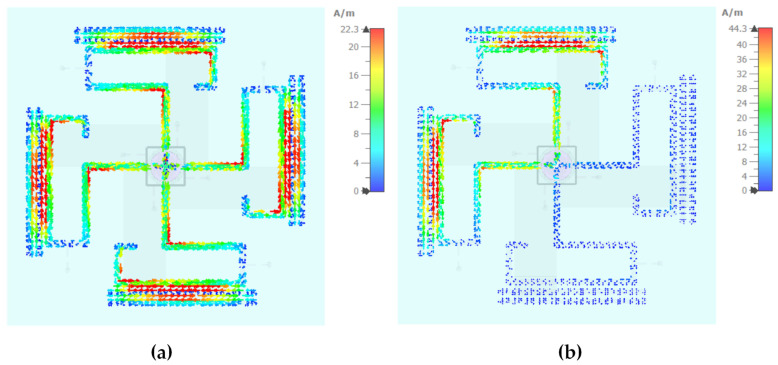
Surface current distribution of the antenna at 5.5 GHz of (**a**) State 1 and (**b**) State 3.

As shown in Figure 4a,b, the surface current distributions of the antenna at 5.5 GHz are presented for State 1 and State 3, respectively. It can be observed that when the antenna operates in State 1, all four PIN diodes are on state, and the antenna exhibits an omnidirectional radiation pattern. In State 3, only diodes 2 and 3 are turned on, and the surface current distribution becomes asymmetric, resulting in a transition of the radiation pattern from omnidirectional to directional, thereby achieving pattern reconfiguration. Except for State 1, the other operating states exhibit similar radiation characteristics to those of State 3. In all states, strong surface currents are concentrated on the hook-shaped radiating patches and the coupling branches, which constitute the main effective radiating region of the antenna.

**Figure 5:**
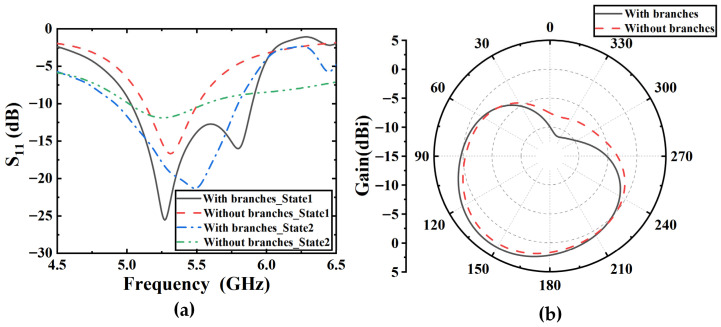
Simulation results of antenna. (**a**) S-parameters of the antenna with and without coupled branches. (**b**) Azimuth radiation pattern of the antenna operating in State 2 with branches and without branches.

Although switching the diode states changes the antenna’s operating mode, the bandwidth remains as narrow as that of a traditional quasi-Yagi antenna. In particular, the bandwidth in omnidirectional mode is only 7.5%. To broaden the bandwidth, coupled branches are introduced near the inverted hook-shaped metal patch. Figure 5a shows the S-parameters of the antenna with and without coupled branches. After introducing the coupled branches, the relative bandwidths of the omnidirectional mode (State 1) and the directional mode (State 2), defined by S_11_ less than −10 dB, reach 16.1% and 16.4%, respectively. Figure 5b shows the azimuth radiation pattern of the antenna with branches and without branches. The main lobe gain is 3.28 dBi with the branches, compared to 2.58 dBi without branches, showing an increase of about 0.7 dBi. In addition, the front-to-back ratio is higher when the branches are present.

**Figure 6:**
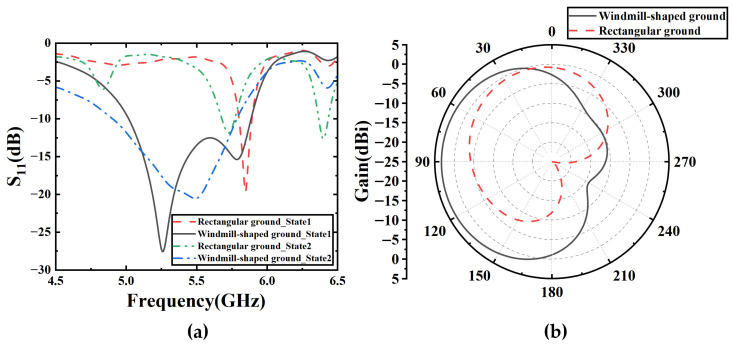
Simulation results of antenna. (**a**) S-parameters of the antenna with rectangular ground and windmill-shaped ground. (**b**) Elevation radiation pattern of the antenna operating in State 2 with rectangular ground and windmill-shaped ground.

Due to the low-profile design characteristics of this structure, the distance between the top-layer radiating patch and the metal ground plane is relatively small, resulting in strong coupling and to some extent affecting the antenna’s downward space radiation capability. To enhance the directionality of the directional mode, a windmill-shaped metal ground was introduced to replace the rectangular metal ground. Figure 6a shows the S-parameters of the antenna with rectangular ground and windmill-shaped ground. It can be seen that the antenna with the windmill-shaped ground exhibits good impedance characteristics, with a common bandwidth of 5.01–5.81 GHz for both omnidirectional and directional modes, where S_11_ is less than −10 dB. In contrast, the square ground degrades the radiation performance. Figure 6b shows the azimuth pattern of the antenna operating in State 2 with windmill-shaped metal ground and rectangular metal ground. The antenna with the windmill-shaped ground has a gain of 3.28 dBi, whereas the antenna with the rectangular ground has a gain of only −0.582 dBi. When the windmill-shaped ground is used, the main lobe is directed at 90°, while with the rectangular ground, the beam points around 30°. This is due to the rectangular ground, which causes the radiation pattern of the antenna to be warped, and the main radiation direction is not on the horizontal plane.

### 3.2. Array Configuration

The proposed low-profile pattern reconfigurable antenna, in the single-element configuration, can achieve azimuth pattern reconfiguration through different combinations of diode switching states. By extending this design to an antenna array, pattern reconfiguration in 3-D full-space can be realized.

**Figure 7:**
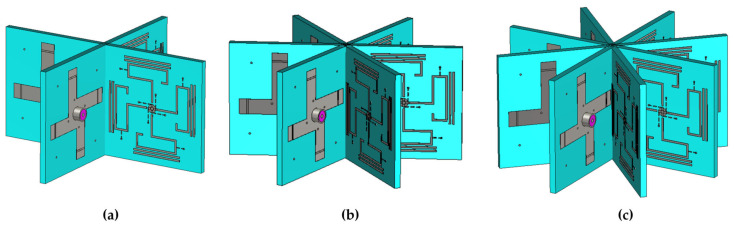
Configurations of arrays with (**a**) four, (**b**) six, and (**c**) eight elements.

**Figure 8:**
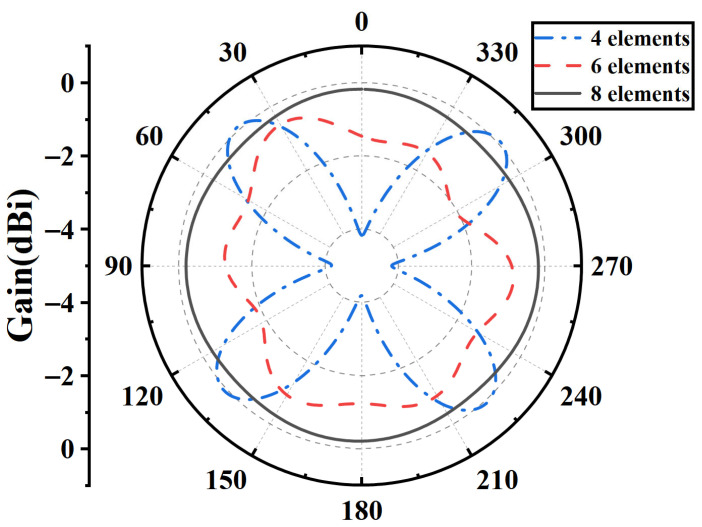
Simulated azimuthal radiation patterns of arrays with four, six, and eight elements.

To determine the optimal number of array elements, comparative simulations were performed for arrays with four, six, and eight elements, with element rotation angles of 90°, 60°, and 45°, respectively. The configurations with four, six, and eight elements correspond to Figure 7a–c. As shown in Figure 8, the azimuthal radiation patterns of the three configurations indicate that increasing the number of elements slightly decreases the horizontal-plane gain but significantly improves the pattern circularity. Considering both the array gain and non-circularity, the eight-element configuration achieves the best overall performance and was therefore adopted in this design.

**Figure 9:**
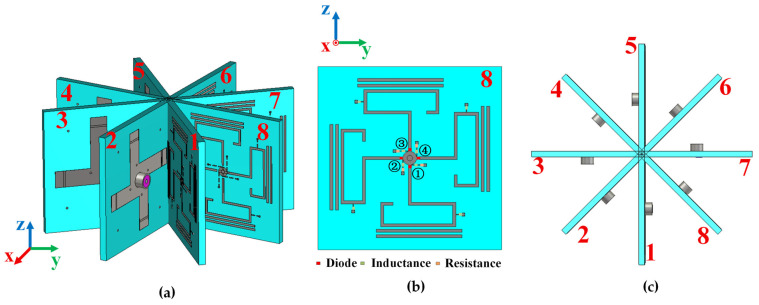
Configuration of the proposed antenna array. (**a**) Perspective view of array. (**b**) Top view of element 8. (**c**) Top view of array.

As shown in Figure 9, the array consists of multiple elements arranged vertically with uniform spacing. This configuration supports switching between omnidirectional and directional modes in the azimuth plane and also enables beam steering in the elevation plane. For clarity, the array elements are numbered and labeled. When all eight elements are simultaneously excited, the array operates in the omnidirectional mode. By controlling the number of excited elements, the array can be switched to the directional mode. As shown in Figure 9b, the top-layer structure of element 8 is presented, where the diodes loaded on it are numbered 1–4. Since the other elements are obtained by rotation, the diode numbers of each element correspond to those of element 8 with respect to the array center.

**Figure 10:**
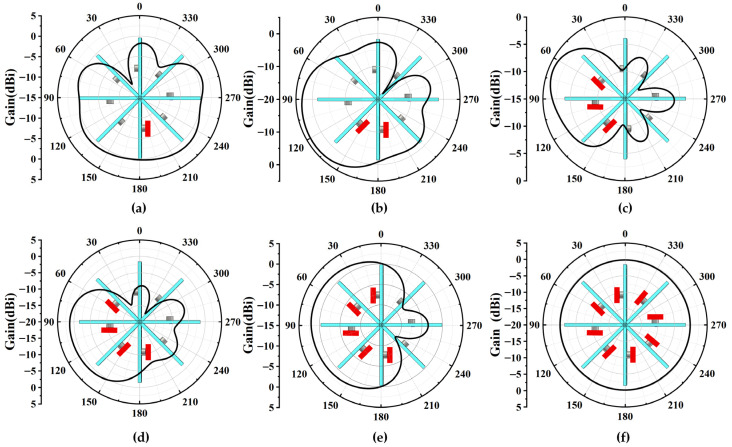
Simulation results of antenna array. (**a**–**f**) Azimuth radiation patterns when different elements are excited.

Figure 10a–f illustrate the azimuth radiation patterns of the antenna array at 5 GHz under the six feeding conditions. The red squares in the figure represent the feeding ports. In all six conditions, only diode 4 on the excited antenna elements is switched to the on state, while the remaining diodes are kept in the off state. Specifically, Figure 10a–e correspond to the five element-excitation combinations, in which one element, two adjacent elements, three adjacent elements, four adjacent elements, and five adjacent elements are excited, respectively, while Figure 10f represents the omnidirectional mode. The optimal radiation performance occurs when the adjacent elements 1 and 2 are excited, achieving a directional gain of 4.17 dBi and a 3 dB beamwidth of 108°. Therefore, the scheme of simultaneously feeding two adjacent elements is selected as the feeding method for pattern reconfiguration. By switching between different pairs of adjacent excited elements, the array can realize multiple distinct directional beams in the entire azimuth plane. Since the array consists of eight elements arranged circularly, feeding each adjacent element pair enables a total of eight discrete directional beams covering the full 360° azimuth range. The eight azimuth beam directions are approximately 30°, 75°, 120°, 165°, 210°, 255°, 300°, and 345°, respectively.

**Figure 11:**
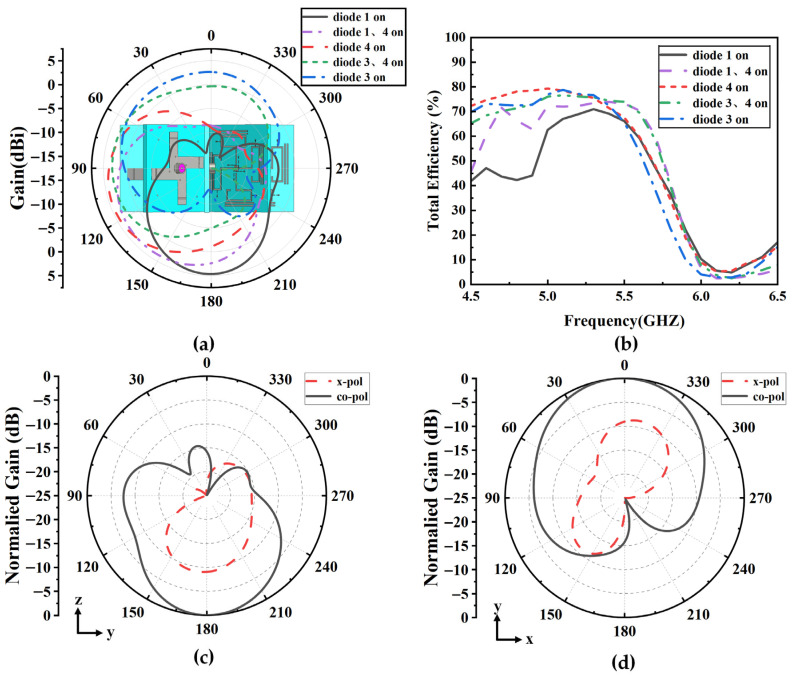
The simulated results of the antenna array when two adjacent elements are excited. (**a**) Elevation radiation patterns with adjacent-element excitation under different diode states. (**b**) Total efficiencies of antenna array. (**c**) Radiation pattern when two adjacent elements are excited and diode 1 is switched on in the *yoz*-plane and (**d**) in the *xoy*-plane.

Figure 11a shows the elevation radiation patterns at 5 GHz when two adjacent elements are simultaneously excited and the diodes on the elements—diodes 1, 3, and 4—are selectively excited, either individually or in pairs. The elevation beams are directed toward 30°, 90°, 120°, 150°, and 180°, respectively, with directional gains greater than 2.9 dBi. The radiation efficiencies of the proposed antenna array are not lower than 40%, as shown in Figure 11b. These results, together with the conclusions drawn from Figure 10, demonstrate that the proposed antenna array supports pattern reconfiguration in 3-D full-space. As an example in which two adjacent elements are excited and diode 1 on the excited elements is switched on, the co-polarized and cross-polarized normalized radiation patterns are shown in Figure 11c,d. Co-pol and x-pol mean co-polarization and cross-polarization, respectively. The cross-polarized component is low, which basically meets the requirements of practical UAV application.

**Figure 12:**
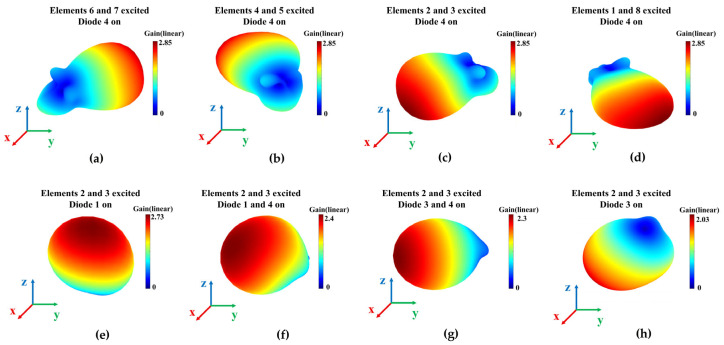
The 3-D radiation patterns of the antenna array. (**a**–**d**) Azimuth-plane radiation pattern reconfiguration; (**e**–**h**) elevation-plane radiation pattern reconfiguration.

To illustrate the 3-D beam coverage of the proposed array, Figure 12 shows several representative 3-D radiation patterns. In Figure 12a–d, four principal azimuth beams at 30°, 120°, 210°, and 300° are plotted; these beams are evenly spaced and are selected from the eight available azimuth directions to demonstrate the 360° coverage of the array. For the elevation plane, when elements 2 and 3 are simultaneously excited, the array can generate five discrete elevation beams at 30°, 90°, 120°, 150°, and 180°, determined by the diode state combinations on the excited elements. Among these, four representative elevation beams are displayed in Figure 12e–h to avoid excessive overlap among the radiation patterns.

## 4. Experimental Results

**Figure 13:**
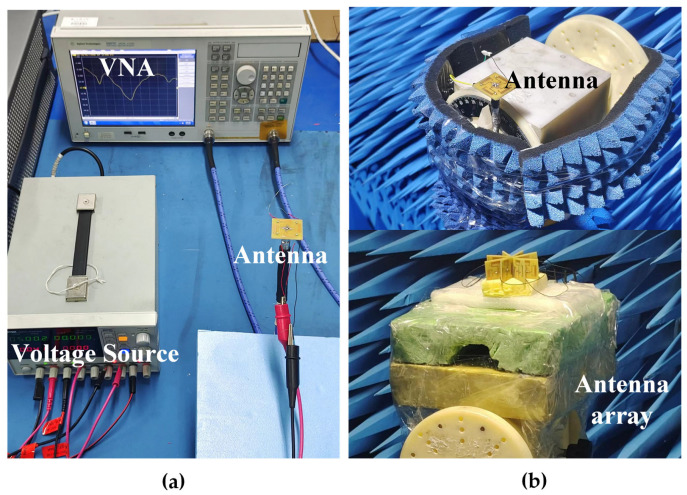
(**a**) S-parameter measurement environment. (**b**) Anechoic chamber environment.

The proposed antenna element and array configuration were fabricated and tested. Figure 13a shows the S-parameters measured setup of the single antenna element. The vector network analyzer (E5071C) is connected to the proposed antenna by coaxial cables. A voltage source is used to supply a stable bias voltage to the diode, ensuring its consistent operation. For a cleaner physical layout of the proposed antenna, the bias line is routed from the top through a metal via to the bottom, where it connects with the wires. Figure 13b presents the radiation pattern testing environment of the single antenna element and the antenna array. The antenna is positioned above the turntable, which undergoes a 360-degree rotation during the testing process to obtain the azimuth radiation pattern. It is crucial to maintain the antenna in a horizontal position during the testing process to avoid inaccurate results. When the antenna array was measured, each antenna element was selectively excited through an 8-way power divider. To minimize the influence of the battery and the power divider on the measured radiation patterns, they were positioned behind the turntable and covered with absorbing material.

**Figure 14:**
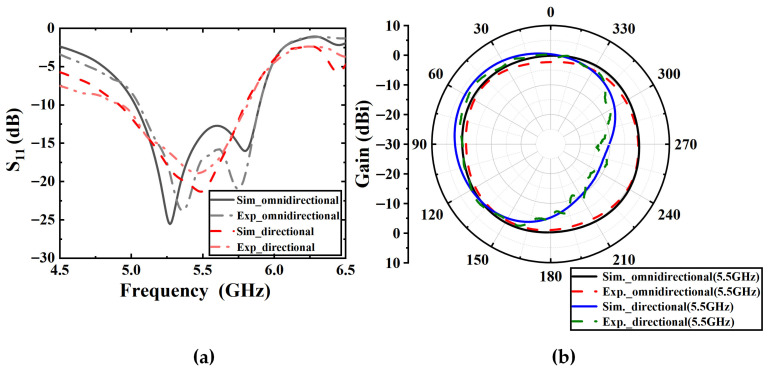
Simulated and measured results of the single antenna element. (**a**) S-parameters. (**b**) Azimuth radiation patterns.

The simulated and measured S-parameters of the single antenna element are shown in Figure 14a, where the measured results show good agreement with simulations. The relative bandwidths of the omnidirectional and directional modes are 16.4% and 16.3%. Due to the variation in surface current distribution, the impedance bandwidths of the omnidirectional and directional modes do not completely overlap, and the common relative bandwidth is 15%. Figure 14b presents the simulated and measured azimuth radiation patterns of the single antenna element at 5.5 GHz. In the omnidirectional mode, the maximum measured gain is 0.5 dBi. In the directional mode, the maximum measured gain is 2.4 dBi. The difference between the measured and simulated results may be caused by fabrication tolerances and the testing environment.

**Figure 15:**
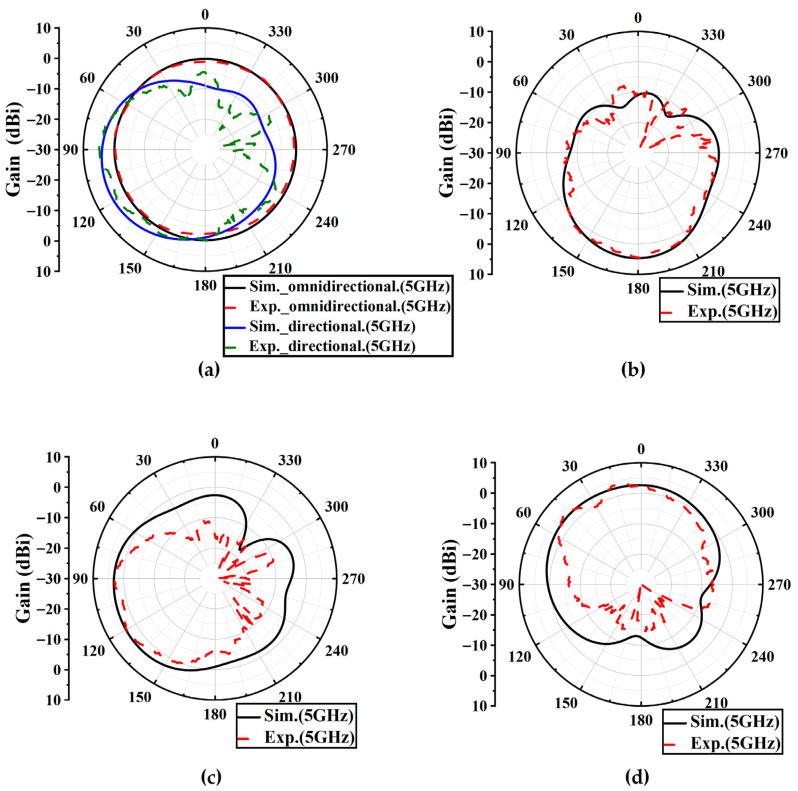
Simulated and measured results of the antenna array. (**a**) Azimuth radiation patterns under all-element excitation and adjacent-element excitation. (**b**–**d**) Elevation radiation patterns under adjacent-element excitation with diode 1, diode 2, and diode 3 in the on state.

The measured azimuth radiation pattern of the antenna array at 5 GHz is presented in Figure 15. In the omnidirectional mode, only diodes 4 on all elements were switched to the on state, while the others were in the off state, and the non-circularity of the pattern is 3.1 dB. The measured results are attributed to manual errors introduced during soldering and element assembly. When two adjacent elements were connected to the power divider while the remaining ports were terminated with 50 Ω loads, only the two adjacent elements were excited, and in this case only diodes 4 on the excited elements were switched on. The measured azimuth radiation pattern at 5 GHz is shown in Figure 15a, where the array operates in the directional mode with a directional gain of 4.9 dBi. Figure 15b–d show the measured elevation radiation patterns when two adjacent elements were excited and diodes 1, 4, and 3 on the excited elements were selectively switched to the on state, with their relative spatial positions referenced to Figure 9. When diode 1 was in the on state, the measurement agreed well with the simulation results. When diodes 4 and 3 were in the on state, the measured main lobe was narrower and the back lobe was lower than the simulation results, indicating better measured directivity.

Table 3 presents a performance comparison between previously reported works and the proposed pattern reconfigurable antenna. Compared with other designs, the proposed antenna offers 3-D full-space coverage radiation reconfiguration capability, while also providing a wider −10 dB bandwidth and more diverse operating modes.

## 5. Conclusions

We propose a pattern reconfigurable quasi-Yagi antenna and array for 3-D full-space coverage. By changing the states of the diodes, the antenna element can flexibly switch between omnidirectional and directional modes. In the omnidirectional mode, the single element operates in 5.02–5.89 GHz, with the maximum gain of 0.5 dBi, and the pattern non-circularity is less than 2 dB. In the directional mode, it operates in 4.93–5.81 GHz, with the maximum gain of 2.4 dBi. In the array configuration, pattern reconfiguration in 3-D full-space can be achieved by controlling the combination of diode states and selecting the excited elements, with a peak gain of 4.9 dBi in the directional modes and a pattern non-circularity of 3.1 dB in the omnidirectional mode. The proposed antenna has the advantages of small size, lightweight, 3-D full-space coverage, and good omnidirectionality, which has potential applications for UAV communication systems.

## Figures and Tables

**Table 1:** Size of the proposed antenna.

Parameters	Value (mm)	Parameters	Value (mm)
*l*_1_	30	*l*_8_	8.5
*l*_2_	6.15	*l*_9_	12.5
*l*_3_	7.25	*D*	2.25
*l*_4_	11.25	*w*_1_	30
*l*_5_	4	*w*_2_	0.5
*l*_6_	2	*w*_3_	4
*l*_7_	14		

**Table 2:** Corresponding combinations of the diodes in different states.

	On	Off
State 1	1, 2, 3, 4	/
State 2	1, 2	3, 4
State 3	2, 3	1, 4
State 4	3, 4	1, 2
State 5	1, 4	2, 3

**Table 3:** Comparison with other works.

Ref.	−10 dB RBW (%)	Operating Modes	3-D Full-Space Coverage	Peak Gain (dBi)
[9]	29.6	D	No	5.9
[12]	22.2	D	No	6
[13]	11	D	No	5
[14]	22.9	OD/D	No	5.3
[16]	2.6	D	No	4.58
[17]	2.45	D	No	7.2
This work	16.4	OD/D	Yes	4.9

OD/D: Omnidirectional/directional. RBW: Relative bandwidth.

## Data Availability

Data are contained within the article.
